# Blood Inflammatory, Hydro-Electrolytes and Acid-Base Changes in Belgian Blue Cows Developing Parietal Fibrinous Peritonitis or Generalised Peritonitis after Caesarean Section

**DOI:** 10.3390/vetsci9030134

**Published:** 2022-03-14

**Authors:** Marie-Charlotte Coenen, Linde Gille, Justine Eppe, Hélène Casalta, Calixte Bayrou, Pierre Dubreucq, Vincent Frisée, Nassim Moula, Julien Evrard, Ludovic Martinelle, Arnaud Sartelet, Philippe Bossaert, Salem Djebala

**Affiliations:** 1 Clinical Department of Production Animals, University of Liège, Quartier Vallée 2, Avenue de Cureghem 7A-7D, 4000 Liège, Belgium; mariecharlotte.coenen@gmail.com (M.-C.C.); linde.gille@uliege.be (L.G.); justine.eppe@uliege.be (J.E.); hcasalta@uliege.be (H.C.); calixte.bayrou@uliege.be (C.B.); pierre.dubreucq@ulg.ac.be (P.D.); vfrisee@uliege.be (V.F.); asartelet@uliege.be (A.S.); 2 Department of Animal Production, University of Liege, Quartier Vallée 2, Avenue de Cureghem 6, 4000 Liège, Belgium; nassim.moula@uliege.be; 3 GIGA Animal Facilities ULiège—B 34, 4000 Liège, Belgium; 4 Gestion et Prévention de Santé, Regional Association of Health and Animal Identification, Allée des Artisans 2, 5590 Ciney, Belgium; julien.evrard@arsia.be; 5 CARE-FEPEX Experimental Station, Faculty of Veterinary Medicine, University of Liège, Quartier Vallée 3, Chemin de la Ferme 6, 4000 Liège, Belgium; lmartinelle@uliege.be; 6 Faculty of Veterinary Medicine, University of Namur, Rue de Bruxelles 61, 5000 Namur, Belgium; philippe.bossaert@unamur.be

**Keywords:** parietal fibrinous peritonitis, generalised peritonitis, caesarean section, inflammatory status, hydration status, acid-base disorders, electrolytes concentration, Belgian blue cattle breed

## Abstract

This study aimed to describe the inflammation, hydro-electrolyte and acid-base imbalances caused by generalised peritonitis (GP) and parietal fibrinous peritonitis (PFP) after caesarean section. After clinical examination, blood was sampled from 11 cows with PFP, 30 with GP and 14 healthy cows. Serum and plasma refractometry and glutaraldehyde tests were used to evaluate the inflammation level, while hydro-electrolytes and acid-base parameters were assessed using an EPOC^®^ device. In addition to clinical signs of dehydration (>10%), blood analysis showed a high fibrinogen concentration (PFP: 8.64 ± 8.82 g/L; GP: 7.83 ± 2.45 g/L) and fast glutaraldehyde coagulation (<3 min) indicative of severe inflammation in both diseases compared to the control group (*p* < 0.05). Moreover, a severe decrease in electrolytes concentration (Na^+^: 126.93 ± 5.79 mmol/L; K^+^: 3.7 ± 1.3 mmol/L; Ca^++^: 0.89 ± 0.12 mmol/L; Cl^−^: 82.38 ± 6.45 mmol/L) and a significant increase in bicarbonate (30.87 ± 8.16 mmol/L), base excess (5.71 ± 7.42 mmol/l), L-lactate (8.1 ± 4.85 mmol/L) and creatinine (3.53 ± 2.30 mg/dL) were observed in cows with GP compared to the control group (*p* < 0.05). In contrast, few major perturbations were noticed in PFP, where only K^+^ (3.64 ± 0.25 mmol/L) and Ca^++^ (1.06 ± 0.09 mmol/L) were significantly modified (*p* < 0.05). In conclusion, a high dehydration and severe inflammation are induced by PFP and GP. Nevertheless, GP causes more electrolytes and acid-base disturbances than PFP.

## 1. Introduction

Generalised peritonitis (GP) and parietal fibrinous peritonitis (PFP) are common complications of caesarean section (CS) [1,2,3,4,5,6]. In fact, GP affects around 4% of cows after CS; its mortality rate is approximately 27% in first line cases [1] and 100% in referral cases [3]. Whilst PFP affects approximatively 1% of cows undergoing CS [1], its mortality rate varies between 8% in the short term and 33% in the long term [3]. Although the prevalence of GP and PFP after CS is still low, the absolute number of these complications is considerable in Belgium [4,5,6], since more than 500,000 CS are performed every year by Belgian vets [7,8,9].

Both GP and PFP are caused by peritoneal inflammation [3]. However, GP is an extensive inflammation of the peritoneal cavity [10,11,12]. The serosa‘s inflammation leads to the perturbation of the peritoneum’s function. In fact, the vasodilatation increases the inflow and the loss of fluids, electrolytes, proteins and inflammatory cells by exudation in the peritoneal cavity [3,11,12,13]. On the other hand, PFP consists of an accumulation of a large volume (5 to 50 L) of inflammatory exudate and fibrin inside a fibrous capsule, localised between the external sheath of parietal peritoneum and the muscular layers of the abdominal wall [2,3,4,5,6,14,15].

Both GP and PFP are triggered by laparotomy. *Trueperella pyogenes* and *Escherichia coli* are the most cultured bacteria in these post-operative complications [2,3,4,5,6,11,12,15]. The anamnesis and clinical signs of both disorders are almost similar. Nevertheless, the symptoms are more severe in GP compared to PFP [3]. The most common clinical signs are pyrexia, dehydration, depression, anorexia, weight loss, abdominal pain and gastrointestinal motility disorders leading to diarrhoea or constipation. These symptoms are the consequence of the compression of digestives organs by a large abdominal mass in the case of PFP and by the accumulation of exudate and fibrin inside of the peritoneal cavity in GP. Furthermore, the clinical signs result from the bacteraemia, endotoxemia and severe inflammatory conditions caused by these illnesses as well [2,3,4,5,6,11,12,15].

The blood hydro-electrolytes and acid-base disturbances are caused by the disorder of the digestive transit and the severe inflammatory status [12,16]. These parameters are frequently described in digestive illnesses [17,18]. They are commonly assessed to administrate supportive fluid therapy and anti-inflammatory drugs to improve the general conditions of patients during surgery, antibiotic therapy and recovery [12,16].

Although GP and PFP disturb the digestive function and escalate the inflammatory status of affected cows [3,15], the hydro-electrolytes, acid-base imbalances and inflammatory conditions resulting in these disorders were not fully described in the scientific literature [2,3,11,12,15,19]. In fact, only succinct descriptions of these parameters were previously reported in PFP and localised peritonitis following a foreign body ingestion [2,15,20,21], but they never reported in the case of GP following CS.

We herein describe the inflammatory conditions, hydro-electrolytes and acid-base disorders caused by PFP and GP after CS in Belgian blue cows. The ultimate goal is to assess the requirement of supportive fluid therapy and anti-inflammatory drugs in combination with the surgical and antimicrobial treatment usually applied to manage these disorders [2,3,6,11,12,15,19].

## 2. Materials and Methods

All procedures received the approval of the Ethical Committee of Liège University (file number 2365).

### 2.1. Animal Selection and Clinical Approach

**Figure 1:**
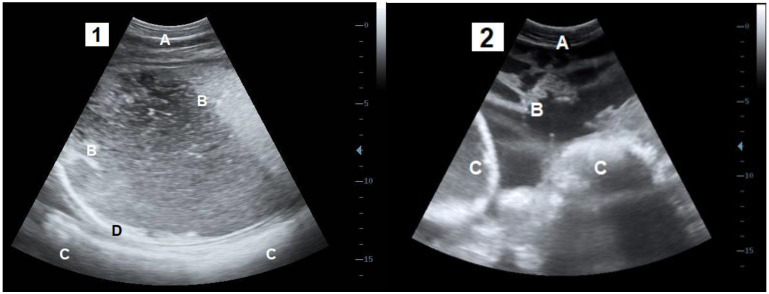
Transabdominal ultrasound sections of the parietal fibrinous peritonitis (**1**) and generalised peritonitis (**2**). A: Abdominal wall; B: Lamellas of fibrin and inflammatory fluids; C: Digestive organs; D: fibrinous capsule.

This study was performed with Belgian blue cows referred to the Veterinary Clinic of Liège University between January 2017 and March 2021. The cows were suspected of PFP or GP by the referring vets based on the anamnesis (recent CS, weight, anorexia and lack of treatment response) and the clinical examination (dehydration, fever (>39°), decrease of ruminal and intestinal motility, restricted arm mobility during rectal palpation) [3]. At arrival, a complete anamnesis was recorded with a special focus on the date of CS (interval CS and the date of the arrival in the clinic) and administrated treatments. The cows that received fluid therapy or cortisone prior to referral were removed from the study. After the clinical examination with a particular focus on the hydration status, based on the skin fold persistence time, the diagnosis of PFP and GP was confirmed by a transabdominal or transrectal ultrasound (3.5 MHz convex probe and 5 MHz linear probe attached to DP-50 Vet^®^, Mindray device, Creteil, France). The specific diagnostic criterion for PFP was the accumulation of an anechogenic fluid and echogenic fibrin strands within a hyperechoic capsule between the parietal sheath of the peritoneum and the muscle layers [2,3,14]. The specific diagnostic criterion for GP was the presence of anechogenic liquids and echogenic fibrin strands within the peritoneal cavity [3,19] (Figure 1). In total, 30 GP and 11 PFP were confirmed.

In order to obtain comparative values of inflammatory, electrolytes and acid-base parameters in healthy individuals, a control group of 14 Belgian Blue cows selected at the experimental farm of Liege University were enrolled. The selection criteria were having calved 1 to 3 months earlier by CS and being in good health. Health status was confirmed by anamnesis (good appetite, no health concerns and no treatment administered since the CS), standard clinical examination (rectal temperature (38–39 °C), respiratory rate (12–36/min), cardiac frequency (40–80/min), dehydration status (no dehydration), buccal, ocular and vulvar mucosa colour (pink), ocular sclera capillary (less than 3 capillaries)) [22] and then by the evaluation of acute and chronic inflammation through serum and plasma refractometry (estimated fibrinogen) and a glutaraldehyde test. The control animals showed less than 6 g/L of blood fibrinogen [23] and a coagulation time of the glutaraldehyde test longer than 15 min [24]. The number of cows needed in the control group was calculated by G* power software [25] using a test strength of 0.80 and a statistical significance threshold of 0.05 (*p* < 0.05)

### 2.2. Sample Collection and Processing

Two 5 mL jugular venous blood were taken in heparinised and plain tubes (Vacutainer, BD). Three ml of plain tube blood were immediately used to perform a glutaraldehyde test [24]. The remainder of the blood without anticoagulant and the heparinised samples were centrifuged (15 min, 1029 g) to assess the fibrinogen concentration. The fibrinogen value was estimated by the difference between the plasma protein (PP) and serum protein (SP) concentrations, established by refractometry (Refractometer^®^, Euromex, Arnhem, The Netherlands) [2,3,26]. Furthermore, 1 mL of jugular blood was sampled in a heparinised syringe to evaluate blood acid-base parameters and electrolytes concentration using an EPOC^®^ blood analyser (Siemens Healthcare, Ottawa, ON, Canada). This device measures and calculates 17 parameters [27,28]. However, the focus in this study was on the acid-base and electrolytes values represented by chemical parameters (sodium (Na^+^), potassium (K^+^), chloride (Cl^−^), ionised calcium (Ca^++^) and anion gap (Agap)), venous blood gas parameters (potential hydrogen (pH), base excess (BE), bicarbonate (HCO_3_^−^) and carbon dioxide pressure (pCO_2_)) and metabolic parameters (glucose, L-lactate and creatinine) [17,23].

### 2.3. Statistical Analysis

The statistical analyses were performed using SAS (2001) (Statistics, Version 8.2. SAS Institute, Cary, NC, USA). Continuous data were checked for normal distribution with a Shapiro-Wilk test. A logarithmic transformation was carried out to convert non-parametric data into parametric data. Descriptive analysis was performed for the time between calving and the date of clinical consultation, the age of cows, inflammatory parameters (SP, PP, fibrinogen), electrolytes and acid-base parameters (Na^+^, K^+^, Cl^−^, Ca^++^, pH, BE, Agap, HCO_3_^−^, glucose, L-lactate and creatinine). The results are presented as a mean ± Standard Deviation (SD).

The means of measured parameters were compared between the different groups of cows (PFP, GP and control group) using analysis of variance (ANOVA).

The test of Pearson was performed to study the correlation between the different variables.

Differences were considered statistically significant at the threshold of *p* < 0.05.

## 3. Results

All the results of the evaluated parameters and statistical analysis are displayed in Table 1 (Supplementary Table S1).

### 3.1. Cows Description

In total, 11 PFP and 30 GP cases were enrolled in the study. All of the patients were Belgian blue cattle breed with a variable age (PFP: range, 24 to 87 months; mean, 46.5 ± 20.07 month; GP: range, 24 to 100 months; mean, 49.44 ± 17.69 months) and parity varying between 1 and 4 CS in both groups. Cows were received in our clinic and diagnosed with these diseases 27.27 ± 12.14 days (12 to 47 days) and 26.16 ± 23.43 days (7 to 90 days) after a CS, respectively, for PFP and GP. The control group consisted of 14 Belgian blue cows calved by CS 1 to 3 months earlier. Their age varied between 28 and 92 months (43.93 ± 21.29 months), with a parity number varying between 1 and 4

### 3.2. Inflammatory Parameters

The total protein concentration rate was statistically superior in PFP cases (SP: 78.45 ± 14.24 g/L; PP: 87.09 ± 11.78 g/L) than in control group cases (SP: 68.36 ± 2.82 g/L; PP: 71.7 ± 3.17 g/L). However, the average protein concentration in GP cases was higher than the value registered in the control group and lower than that recorded in PFP cases, but no statistical difference was observed.

Results of the blood inflammatory parameters indicated a severe inflammatory status in sick cows. Indeed, the fibrinogen concentration was statistically higher in cows affected by PFP (8.64 ± 4.82 g/L) and those affected by GP (7.83 ± 2.45 g/L) compared to the healthy group (3.36 ± 0.93 g/L). Furthermore, a significant difference was noticed in the coagulation times of the glutaraldehyde test, since the complete coagulation was obtained in less than three minutes in all of the patients, while no coagulation was observed after 15 min in the healthy group.

### 3.3. Hydro-Electrolyte and Acid-Base Parameters

#### 3.3.1. Clinical Evaluation of the Hydration Status

Independently of the illness, all of the patients showed an infinite persistent skin folder test, indicating a dehydration level higher than 10%. On the contrary, the control group showed a normal hydration status, with a skin fold persistence of less than two seconds [22,23].

#### 3.3.2. Chemical Parameters

Overall, a decrease of Na^+^, K^+^, Cl^−^ and Ca^++^ were observed in the sick cows compared to the control group. However, the decrease of these parameters was more severe in cows affected by GP compared to those developing PFP. Indeed, only K^+^ (3.64 ± 0.25 mmol/L) and Ca^++^ (1.06 ± 0.09 mmol/L) significantly declined in PFP cases compared to the control group. In contrast, the diminution of the electrolytes concentration (Na^+^: 126.93 ± 5.79 mmol/L; K^+^: 3.7 ± 1.3 mmol/L; Ca^++^: 0.89 ± 0.12 mmol/L; Cl^−^: 82.38 ± 6.45 mmol/L) in cases of GP was sharper and statistically significant compared to the control group (Na^+^: 133.14 ± 2.21 mmol/L; K^+^: 4.48 ± 0.42 mmol/L; Ca^++^: 1.19 ± 0,04 mmol/L; Cl^−^: 96.93 ± 2.73 mmol/L). The Agap average concentration was almost the same in PFP cases as in the control group. However, a statistically higher Agap concentration was observed in cows with GP (18.82 ± 5.56 mmol/L) compared to the healthy group (15.28 ± 1.86 mmol/L).

#### 3.3.3. Venous Blood Gas

A mild increase in measured venous blood gas parameters was observed in cows with PFP compared to those from the control group, but no statistical difference was shown. The same observation could be stated regarding the cows with GP, except for HCO_3_^−^ (30.87 ± 8.16 mmol/L) and BE (5.71 ± 7.42 mmol/L), in which the increases were sharper and statistically significant compared to the control group (HCO_3_^−^: 25.56 ± 1.86 mmol/L; BE: 1.06 ± 1.51 mmol/L).

#### 3.3.4. Metabolic Parameters

Although the average concentrations of glucose, L-lactate and creatinine were superior in PFP cows compared to the control group, the difference was statistically non-significant. Nevertheless, the increase of L-lactate (8.1 ± 4.85 mmol/L) and creatinine (3.53 ± 2.30 mg/dL) in the group of GP was statistically significant compared to the control cows (L-lactate: 1.68 ± 1.44 mmol/L; creatinine: 2.18 ± 0.28 mg/dL). Furthermore, the average L-lactate concentration was significantly superior in GP cows compared to those with PFP. The glucose concentration in GP and PFP cows was higher than the average value seen in the control group, but no statistical difference was observed.

### 3.4. The Correlation between the Different Parameters

The evaluation of the interaction among the different parameters has revealed a negative correlation between the concentration of BE and Cl^−^ (r = −0.45; *p* < 0.05) and between the Na^+^ and fibrinogen (r = −0.37; *p* < 0.05) in cows affected with GP. Moreover, a positive correlation was observed between the L-lactate and Agap concentrations in all of the cows involved in this study.

## 4. Discussion

Despite the important amount of PFP and GP in the Belgian field veterinary practice following CS, there is a lack of scientific knowledge about the disorders caused by these post-operative complications [2,3,4,6]. This study presents a unique dataset of inflammatory status, acid-base and electrolytes perturbations in cows suffering from PFP and GP following CS. Moreover, the comparison between healthy and sick cows makes the study more relevant. Unfortunately, the findings would be more accurate if the cows of the control group came from the same farms and had exactly the same interval between the CS and the day of sampling as the sick cows. However, due to practical reason, it was not possible to have identical matches.

The assessment of inflammatory parameters allowed us to choose healthy cows for the control group and quantify the level of inflammation in cows with PFP and GP. Sick cows showed a severe inflammatory status compared to the control cows [2,3,15], as shown by the high fibrinogen concentration and the speed of glutaraldehyde coagulation in cows with CS complications compared to those of the control group [23,24].

The total proteins concentrations (SP, PP) were higher in cows with PFP than in cows from the control group. This is caused by the increased production of inflammatory proteins such as fibrinogen and Ɣ-globulin, further escalated by the high dehydration status (>10%) highlighted in this group [2,3,15]. Nevertheless, the average proteins concentration in cows with GP was not significantly different compared to the mean of the control group in spite of the high level of inflammatory proteins and extreme dehydration. This could be explained by an abundant loss of proteins through the extensive inflammation of the peritoneal serosa [3,11,12]. In fact, cows affected by GP could even show a hypoproteinaemia when they had a normal hydration status [11,23,30]. This high level of inflammation would explain several clinical symptoms of PFP and GP such as fever, anorexia, dehydration and reduced intestinal motility [2,3,5,11,31].

The assessment of the inflammatory status could have been even more delineated if we had performed the electrophoresis of total proteins. This would give us more information on the proteins’ composition, especially regarding the albumin and globulin fraction [20,23].

Blood acid-base balance and electrolyte values were assessed using the EPOC^®^ device (Siemens Healthcare, Ottawa, ON, Canada). This blood analyser is very convenient; since it is transportable, it could be used on the cow side. Nevertheless, this device has never been validated for ruminants [27]. Although it is used in several species (dogs, horses, alpaca and lama), the scientific literature about the consistency of this device is debatable [27,28,32,33,34]. The lack of a reference range for the assessed parameters is one of the weaknesses of this tool [27,28]. However, in our study, the ranges of values found in the control group are very close to the reference ranges stated by Carlson, 2009 [29].

Blood electrolytes imbalance is often caused by anorexia, absorption disorders or ions sequestration in the digestive system. Overall, the ions H^+^ and Cl^−^ are produced in the abomasum and released in the small bowels, while Na^+^, K^+^ and Ca^++^ come from the food ingestion [16]. The decline in the ions concentration observed in GP results from the digestive ileus following fibrine and exudate accumulation in the peritoneal cavity [3,11,35], reducing the Cl^−^ and H^+^ production and leading to the trapping of these ions in the abomasum. Moreover, anorexia caused by the inflammatory status [3,11] reduces the supply and the absorption of electrolytes inducing hyponatremia, hypokalemia and hypocalcemia. In fact, a negative correlation between fibrinogen and Na^+^ was observed in GP.

In contrast, cows with PFP showed a significant decrease only for Ca^++^ and K^+^; this is a direct consequence of anorexia [2,3,15]. However, Cl^−^ concentration was not significantly modified, since the digestive transit was still present but slowed down [2,3,15]. The presence of intestinal motility may explain the mild hyponatremia in cows with PFP. In fact, even if appetite is low, the limited Na^+^ ingested is absorbed at the intestinal level.

The blood ions perturbations directly impact the acid-base equilibrium and vice-versa [16]. According to the simplified theory of Stewart, alkalosis is defined as an increase in strong ion difference, a decrease in pCO_2_ and a decrease in non-volatile weak acids buffers such as proteins and phosphorus [18]. Overall, our results showed that the acid-base equilibrium tend to alkalosis in cows with GP, since BE is significantly higher in this group. This is mainly related to the sequestration of Cl^−^ and H^+^ in the digestive system. Indeed, a negative correlation was observed between BE and Cl^−^ in this group of cows. The decrease of Cl^−^ leads to the decline of strong ions difference, inducing metabolic alkalosis. Moreover, the decrease of H^+^ highlighted by the value of pH induced the accumulation HCO_3_^−^, which is supposed to be combined with H^+^ and eliminated through the respiratory way [18,36]. The pCO_2_ was within the normal range in both groups, indicating a lack of respiratory acidosis or alkalosis [18,36].

None of these parameters (BE, HCO_3_^−^, pCO_2,_ pH) were significantly modified in cows with PFP, probably due to the digestive perturbations caused by PFP being less important than those induced by GP [3].

The Agap is an estimation of the unmeasurable plasmatic anions, of which the most important are ionised albumin, phosphor, ketone and sulphate. It allows for the specification of another origin of acid-base imbalance [18,37]. The Agap’s value is higher when the concentration of the unmeasured anions increases [37,38]. The average Agap is significantly higher in cows with GP than in the others. Indeed, the high L-lactate concentration could be the principal cause of the Agap surge, since a positive correlation was observed between these two elements. The level of L-lactate indicates an increase in anaerobic metabolism [13,38], which is caused by the dehydration, inflammation and severe compression of digestives organs by the fibrin accumulation in cows with GP [3,11].

The increase in creatinine concentration due to the acute prerenal renal failure confirmed a high level of dehydration observed in all of the patients [3,23]. Furthermore, it shows that cows with GP are more dehydrated than those affected by PFP [23], although no difference in dehydration status was clinically noticed. The glucose concentration is almost the same in the different groups, even though it tended to be higher in GP cows. This could be relative to the catecholamine liberation following the stress and the abdominal pain [3,11,15].

All of these modifications need to be considered during the treatment of these disorders. In fact, based on our study, it seems more than indicated to use supportive fluid therapy either by intravenous or oral ways and anti-inflammatory drugs during the treatment of PGP and GP. Moreover, Cl^−^, L-lactate and Agap concentrations are frequently used as predictive values of treatment, resulting in digestive disorders [17,39,40], and they could explain the poor prognosis of cows with GP [3].

## 5. Conclusions

Our study elucidates the blood inflammation, acid-base and electrolyte imbalances present in Belgian Blue cows developing GP and PFP after CS. Indeed, severe dehydration, high inflammatory status (fibrinogen and glutaraldehyde test) and huge modifications of chemical (Na^+^, K^+^, Cl^−^, Ca^++^ and Agap), blood gas (HCO_3_^−^ and BE) and metabolic (L-lactate and creatinine) parameters were noticed in cows with GP. On the contrary, the perturbations induced by PFP are milder and touch only the hydration status and the inflammatory (fibrinogen and glutaraldehyde test) and some of the chemical parameters (K^+^ and Ca^++^). All of these modifications should be taken into consideration during the treatment of PFP and GP.

## Figures and Tables

**Table 1:** Results (mean ± standard deviation and range) and a comparison of the evaluated parameters (inflammatory, chemical, venous blood gas and metabolic parameters) in cows developing parietal fibrinous peritonitis, cows developing generalised peritonitis and the control group.

Inflammatory Parameters		PFP (11 Cows)	GP (30 Cows)	Control Group (14 Cows)	Reference Values [29]
SP (g/L)	Mean ± SD	78.45 ± 14.24 ^a^	71.33 ± 13.26 ^ab^	68.36 ± 2.82 ^b^	68–86
	Range	58–100	40–99	64–74	
PP (g/L)	Mean ± SD	87.09 ± 11.78 ^a^	78.97 ± 14.15 ^ab^	71.7 ± 3.17 ^b^	No reference value
	Range	66–106	46–107	67–78	
Fibrinogen (g/L)	Mean ± SD	8.64 ± 4.82 ^a^	7.83 ± 2.45 ^a^	3.36 ± 0.93 ^b^	1–6
	Range	0–18	4–12	2–5	
glutaraldehyde test (min)		<3	<3	>15	>15
**Chemical parameters**					
Na^+^ (mmol/L)	Mean ± SD	130.9 ± 5.57 ^a^	126.93 ± 5.79 ^b^	133.14 ± 2.21 ^a^	132–152
	Range	122–137	116–137	130–137	
K^+^ (mmol/L)	Mean ± SD	3.64 ± 0.25 ^a^	3.7 ± 1.3 ^a^	4.48 ± 0.42 ^b^	3.9–5.8
	Range	3.2–3.9	2.1–5.4	4.2–5.2	
Ca^++^ (mmol/L)	Mean ± SD	1.06 ± 0.09 ^a^	0.89 ± 0.12 ^b^	1.19 ± 0.04 ^c^	0.97–1.24
	Range	0.89–1.16	0.5–1.1	1.09–1.26	
Cl^−^ (mmol/L)	Mean ± SD	93 ± 6.15 ^a^	82.38 ± 6.45 ^b^	96.93 ± 2.73 ^a^	97–111
	Range	79–101	65–95	92–102	
Agap (mmol/L)	Mean ± SD	15 ± 4.82 ^a^	18.82 ± 5.56 ^b^	15.28 ± 1.86 ^a^	14–20
	Range	10–24	10–30	12–20	
**Venous blood gas parameters**					
pH	Mean ± SD	7.73 ± 0.07 ^a^	7.47 ± 0.08 ^a^	7.42 ± 0.07 ^a^	7.31–7.53
	Range	7.28–7.52	7.31–7.76	7.31–7.52	
HCO_3_^−^ (mmol/L)	Mean ± SD	26.47 ± 4.15 ^a^	30.87 ± 8.16 ^b^	25.56 ± 1.86 ^a^	0–6
	Range	19.2–34.6	14–50.8	20.6–28.6	
pCO_2_ (mmHg)	Mean ± SD	40.03 ± 6.29 ^a^	40.94 ± 6.8 ^a^	39.76 ± 8.02 ^a^	35–45
	Range	31.9- 51.3	25.6- 53.5	31.8–52.2	
BE (mmol/L)	Mean ± SD	1.98 ± 4.35 ^a^	5.71 ± 7.42 ^b^	1.06 ± 1.51 ^a^	21–32
	Range	−5.7–9.3	−10.29–23.7	−2.3–2.9	
**Metabolic parameters**					
Glucose (mg/dL)	Mean ± SD	80.36 ± 33.31 ^a^	86.13 ± 39.32 ^a^	74 ± 5.76 ^a^	33–66
	Range	42–165	43–213	64–82	
L-lactate (mmol/L)	Mean ± SD	4.68 ± 4.82 ^a^	8.1 ± 4.85 ^b^	1.68 ± 1.44 ^a^	0.56–2.22
	Range	0.7–16.19	1.14–19.03	0.43–4.97	
Creatinine (mg/dL)	Mean ± SD	2.33 ± 1.15 ^ab^	3.53 ± 2.30 ^a^	2.18 ± 0.28 ^b^	1.81–2.84
	Range	0.97–4.34	1.22–11.46	1.7–2.72	

PFP: parietal fibrinous peritonitis; GP: generalised peritonitis SP: serum protein; PP: plasma protein; Na^+^: sodium; K^+^: potassium; Cl^−^: chloride, Ca^++^: ionised calcium; (Agap) anion gap; pH: potential hydrogen, BE: base excess; HCO_3_^−^: bicarbonate; pCO_2_: carbon dioxide pressure. Within the same line, values bearing a distinct letter are statistically different from each other (*p* < 0.05).

## Data Availability

All data are available in the manuscript.

## Supplementary Materials

The following supporting information can be downloaded at: https://www.mdpi.com/article/10.3390/vetsci9030134/s1, Table S1: Results (mean ± standard deviation) and comparison of the evaluated parameters (inflammatory, chemical, venous blood gas, and metabolic parameters) in cows developing parietal fibrinous peritonitis, generalised peritonitis and the control group.
