# Supplementary material for: Blood Inflammatory, Hydro-Electrolytes and Acid-Base Changes in Belgian Blue Cows Developing Parietal Fibrinous Peritonitis or Generalised Peritonitis after Caesarean Section
Source: Vet Sci. 2022 Mar 14;9(3):134. doi: 10.3390/vetsci9030134 (PMC8949059; doi:10.3390/vetsci9030134)
Supplement: Supplementary file 1 [file vetsci-09-00134-s001.zip › vetsci-1607221-supplementary.pdf]

Table S1. Results (mean ± standard deviation) and comparison of the evaluated parameters (inflammatory, chemical, venous blood gas, and metabolic parameters) in cows developing parietal fibrinous peritonitis, generalised peritonitis and the control group.

**Inflammatory parameters**		PFP (11 cows)	Control group (14 cows)	*p*-Value	GP (30 cows)	Control group (14 cows)	*p*-Value	PFP (11 cows)	GP (30 cows)	*p*-Value
SP (g/l)	Mean ± SD	78.45±14.24 ^{a}	68.36±2.82 ^{b}	0.0383	71.33±13.26 ^{a}	68.36±2.82 ^{a}	0.4702	78.45±14.24 ^{a}	71.33±13.26 ^{a}	0.0840
PP (g/l)	Mean ± SD	87.09±11.78 ^{a}	71.7±3.17 ^{b}	0.0022	78.97±14.15 ^{a}	71.7±3.17 ^{a}	0.0647	87.09±11.78 ^{a}	78.97±14.15 ^{a}	0.0576
Fibrinogen (g/l)	Mean ± SD	8.64±4.82 ^{a}	3.36±0.93 ^{b}	0.0001	7.83±2.45 ^{a}	3.36±0.93 ^{b}	0.0001	8.64±4.82 ^{a}	7.83±2.45 ^{a}	0.4253
glutaraldehyde test (min)		< 3	> 15	0.0001	< 3	> 15	0.0001	< 3	< 3	0.1035
		**Chemical parameters**								
Na^{+} (mmol/L)	Mean ± SD	130.9±5.57 ^{a}	133.14±2.21 ^{a}	0.2800	126.93±5.79 ^{a}	133.14±2.21 ^{b}	0.0004	130.9±5.57 ^{a}	126.93±5.79 ^{b}	0.0314
K^{+} (mmol/L)	Mean ± SD	3.64±0.25 ^{a}	4.48±0.42 ^{b}	0.0393	3.7±1.3 ^{a}	4.48±0.42 ^{b}	0.0188	3.64±0.25 ^{a}	3.7±1.3 ^{a}	0.8496
Ca^{++} (mmol/L)	Mean ± SD	1.06±0.09 ^{a}	1.19±0.04 ^{b}	0.0033	0.89±0.12 ^{a}	1.19±0.04 ^{b}	0.0001	1.06±0.09 ^{a}	0.89±0.12 ^{b}	0.0001
Cl^{-} (mmol/L)	Mean ± SD	93±6.15 ^{a}	96.93±2.73 ^{a}	0.0918	82.38±6.45 ^{a}	96.93±2.73 ^{b}	0.0001	93±6.15 ^{a}	82.38±6.45 ^{b}	0.0001
Agap (mmol/L)	Mean ± SD	15±4.82 ^{a}	15.28±1.86 ^{a}	0.8811	18.82±5.56 ^{a}	15.28±1.86 ^{b}	0.0263	15±4.82 ^{a}	18.82±5.56 ^{b}	0.0271
		**Venous blood gas parameters**								
pH	Mean ± SD	7.73±0.07 ^{a}	7.42±0.07 ^{a}	0.8681	7.47±0.08 ^{a}	7.42±0.07 ^{a}	0.0708	7.73±0.07 ^{a}	7.47±0.08 ^{a}	0.1390
HCO_3^{-} (mmol/L)	Mean ± SD	26.47±4.15 ^{a}	25.56±1.86 ^{a}	0.7272	30.87±8.16 ^{a}	25.56±1.86 ^{b}	0.0137	26.47±4.15 ^{a}	30.87±8.16 ^{b}	0.0476
pCO_2 (mmHg)	Mean ± SD	40.03±6.29 ^{a}	39.76±8.02 ^{a}	0.9241	40.94±6.8 ^{a}	39.76±8.02 ^{a}	0.6090	40.03±6.29 ^{a}	40.94±6.8 ^{a}	0.7179
BE (mmol/L)	Mean ± SD	1.98±4.35 ^{a}	1.06±1.51 ^{a}	0.7015	5.71±7.42 ^{a}	1.06±1.51 ^{b}	0.0187	1.98±4.35 ^{a}	1.06±1.51 ^{a}	0.0794
		**Metabolic parameters**								
Glucose (mg/dL)	Mean ± SD	80.36±33.31 ^{a}	74±5.76 ^{a}	0.6334	86.13±39.32 ^{a}	74±5.76 ^{a}	0.2601	80.36±33.31 ^{a}	86.13±39.32 ^{a}	0.6211
L-lactate (mmol/L)	Mean ± SD	4.68±4.82 ^{a}	1.68±1.44 ^{a}	0.0865	8.1±4.85 ^{a}	1.68±1.44 ^{b}	0.0001	4.68±4.82 ^{a}	8.1±4.85 ^{b}	0.0268
Creatinine (mg/dL)	Mean ± SD	2.33±1.15 ^{a}	2.18±0.28 ^{a}	0.8382	3.53±2.30 ^{a}	2.18±0.28 ^{b}	0.0240	2.33±1.15 ^{a}	3.53±2.30 ^{a}	0.0621

PFP: parietal fibrinous peritonitis; GP: generalised peritonitis; SP: serum protein; PP: plasma protein; Na^{+}: sodium; K^{+}: potassium; Cl^{-}: chloride, Ca^{++}: ionized calcium; (Agap) anion gap; pH: potential hydrogen, BE: base excess; HCO_3^{-}: bicarbonate; pCO_2: carbon dioxide pressure. Within the same line, values bearing a distinct letter are statistically different from each other ($p<0.05$).
